# Transmission of Respiratory Viral Diseases to Health Care Workers: COVID-19 as an Example

**DOI:** 10.1146/annurev-publhealth-052120-110009

**Published:** 2022-01-07

**Authors:** Amanda M. Wilson, Darrah K. Sleeth, Camie Schaefer, Rachael M. Jones

**Affiliations:** 1 Department of Family and Preventive Medicine, Spencer Fox Eccles School of Medicine, University of Utah, Salt Lake City, Utah, USA; 2 Department of Community, Environment and Policy, Mel and Enid Zuckerman College of Public Health, The University of Arizona, Tucson, Arizona, USA

**Keywords:** respiratory virus, COVID-19, health care worker, aerosol, occupational health

## Abstract

Health care workers (HCWs) can acquire infectious diseases, including coronavirus disease 2019 (COVID-19), from patients. Herein, COVID-19 is used with the source–pathway–receptor framework as an example to assess evidence for the roles of aerosol transmission and indirect contact transmission in viral respiratory infectious diseases. Evidence for both routes is strong for COVID-19 and other respiratory viruses, but aerosol transmission is likely dominant for COVID-19. Key knowledge gaps about transmission processes and control strategies include the distribution of viable virus among respiratory aerosols of different sizes, the mechanisms and efficiency by which virus deposited on the facial mucous membrane moves to infection sites inside the body, and the performance of source controls such as face coverings and aerosol containment devices. To ensure that HCWs are adequately protected from infection, guidelines and regulations must be updated to reflect the evidence that respiratory viruses are transmitted via aerosols.

## INTRODUCTION

The coronavirus disease 2019 (COVID-19) pandemic has adversely impacted the health and well-being of health care workers (HCWs) in myriad ways, including through occupationally acquired infection. Through July 16, 2020, approximately 100,000 COVID-19 cases reported to the US Centers for Disease Control and Prevention (CDC) were identified as HCWs, comprising 14% of cases for which health care worker status was reported (40). Though it is not possible to know how many of these infections arose through workplace exposures, COVID-19 prevalence has been reported to be significantly higher among HCWs than non-HCWs (9).

COVID-19, however, is simply the latest viral respiratory infectious disease to adversely impact the occupational health of HCWs. The 2003–2004 pandemic of severe acute respiratory syndrome coronavirus (SARS-CoV) was recognized for high rates of infection and mortality among HCWs (91). The occupational health burden of endemic diseases among HCWs is difficult to know, owing to the lack of surveillance and the difficulty of attributing disease to occupational exposure. Risk assessment methodology can provide some insight. With respect to seasonal influenza, for example, a risk assessment found that HCWs in acute care settings have frequent occupational exposures and incur occupational influenza infections at rates similar to community rates (48, 49).

The primary objective of this article is to review evidence regarding the transmission of viral respiratory infections to HCWs from patients, using COVID-19 as the primary example. Two transmission processes are considered: aerosol transmission and indirect contact transmission. Aerosol transmission is used because, as defined by Jones & Brosseau (46), it encompasses all transmission processes that involve the transport of pathogens through air, including droplet, short-range airborne, and airborne transmission (60, 101). Indirect contact transmission, which involves virus-contaminated surfaces (e.g., fomites) as an intermediary between the patient and HCWs (15), is a commonly recognized transmission route for viral respiratory infectious diseases (101). The secondary objectives of this article are to discuss the implications of the transmission processes for control strategies to protect HCWs and to identify knowledge gaps to prioritize future research.

The transmission processes of COVID-19 to HCWs and among the public have been contentiously debated. Early in the COVID-19 pandemic, the US CDC and the World Health Organization (WHO) communicated that COVID-19 was transmitted through the spray of respiratory droplets and indirect contact transmission to the facial mucous membranes. This information was challenged, with many arguing that inhalation of severe acute respiratory syndrome coronavirus 2 (SARS-CoV-2) could result in infection (73, 100). In April 2021, the WHO updated their description of COVID-19 transmission, writing that “[a] person can be infected when aerosols or droplets containing the virus are inhaled or come directly into contact with the eyes, nose or mouth” (112). The US CDC had been more equivocal but in May 2021 wrote that COVID-19 is transmitted by exposure to infectious respiratory fluids, most commonly by “inhalation of very fine respiratory droplets and aerosol particles” (19). This change has been accompanied by a deemphasis of indirect contact transmission (19).

## DEFINITIONS

Vocabulary, including the collapse of multiple meanings into a single term, has been a barrier to discussing viral respiratory disease transmission. This situation has arisen because multiple disciplines are engaged in the study of respiratory infectious diseases, and respiratory virus transmission is highly complex, involving multiple spatial scales, media, emission mechanisms, and infection sites (46, 60, 101). Definitions used herein are provided in the margins. The phrases “small-particle aerosols” and “droplet nuclei” refer to particles of a size similar to that of respirable particles (42, 101).

## SOURCE–PATHWAY–RECEPTOR FRAMEWORK

**Figure 1:**
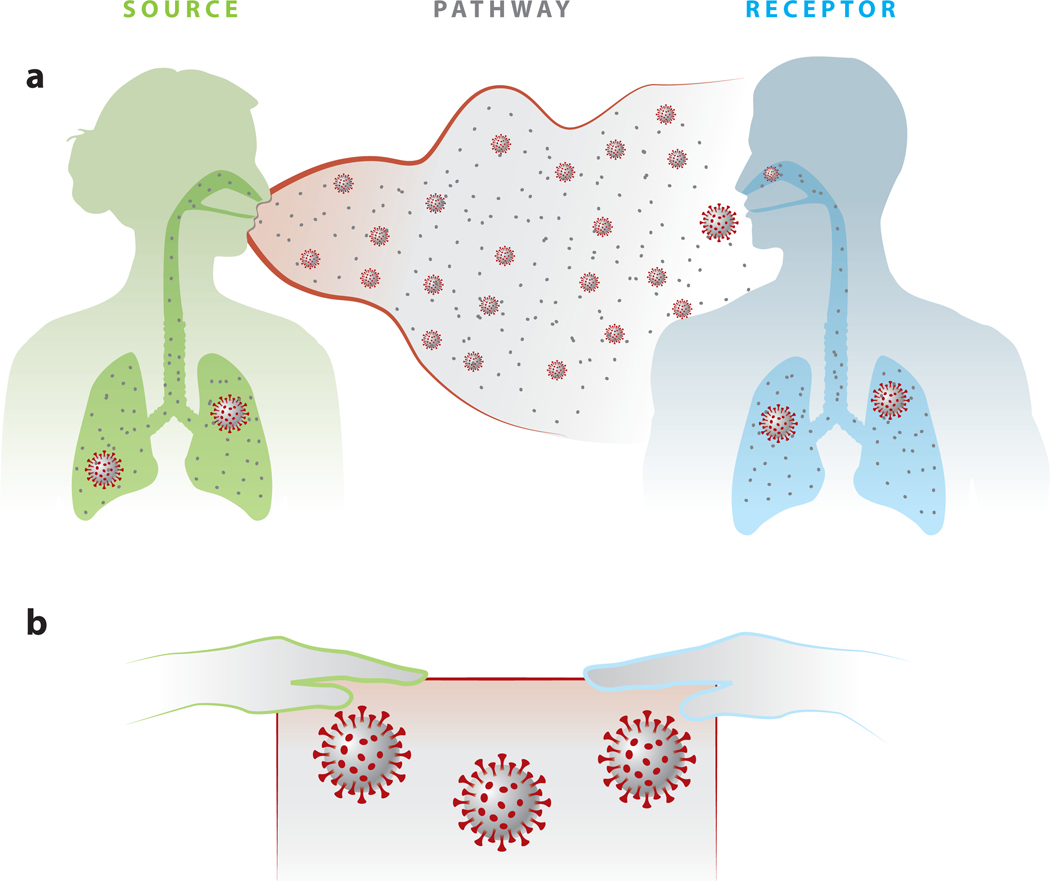
Simplified depiction of the source (infected person, *green*), pathway (environment, *gray*), and receptor (susceptible person, *blue*) model for coronavirus disease 2019 (COVID-19). Pathway shown as airborne (panel *a*) and across surfaces (panel *b*).

Evidence regarding the transmission of viral respiratory infections is presented following the source–pathway–receptor framework, a framework commonly used in occupational and environmental health to describe the movement of pollutants through a system. As shown in Figure 1, the pollutant of concern herein is respiratory viruses, and the system is a health care setting containing a source (infectious patient) and a receptor (susceptible HCW). The pathways are the processes by which the pathogen moves from the source to the receptor, such as air and fomites. This framework has been utilized previously to assess the evidence for aerosol-transmissible diseases (46). Table 1 presents questions that must be answered to demonstrate the feasibility of a transmission process using the source–pathway–receptor framework.

## EVIDENCE FOR AEROSOL TRANSMISSION

### Source

For a virus to cause an aerosol-transmissible disease, it must be aerosolized upon emission from the source. Natural biological processes—breathing, talking, coughing, etc.—generate aerosols of respiratory secretions (2, 23), and aerosols can be formed during some medical procedures (108, 117). But to transmit disease, generated aerosols must contain infectious pathogens.

Owing to the presence of viable SARS-CoV-2 in respiratory secretions (114), one can expect that viable virus could be emitted from the respiratory tract. SARS-CoV-2 RNA has been found in exhaled breath of COVID-19 cases (67), but viability was not assessed. SARS-CoV-2 RNA is routinely found in the air around hospitalized COVID-19 patients, particularly in intensive care units (12), and a few studies have cultured SARS-CoV-2. One study detected viable SARS-CoV-2 in the hospital rooms of two COVID-19 patients with stationary samplers located 2–4.8 m from patients (57), and another study collected a sample that was suggestive of viable virus in the hall-way outside a COVID-19 patient room (97). Overall, these results indicate that COVID-19 cases can emit infectious SARS-CoV-2 in aerosol, which is consistent with SARS-CoV-2 transmission among animals in the absence of physical contact (22, 93).

The size of infectious aerosols emitted by the source influences the pathway and exposure of the receptor. To date, limited research has been done on the size distribution of infectious aerosols. Viable SARS-CoV-2 measured around COVID-19 patients was likely in respirable particles, owing to the distance between the patient and the sampler and the design of the sampler (57, 97). SARS-CoV-2 RNA has been detected in the air of patient rooms in three size ranges: <1 μm, 1–4 μm, and >4 μm (25, 80). While SARS-CoV-2 RNA has been found in particles >4 μm, these studies provided no further information about the particle size distribution, and the air sampling method and strategy were unlikely to capture virus in droplet spray. Thus, existing data indicate that infectious SARS-CoV-2 can be present in respirable aerosols, and while infectious SARS-CoV-2 is likely also present in larger particles involved in droplet transmission, this assumption has not been demonstrated to date.

The evidence of SARS-CoV-2 emission in respiratory aerosols is consistent with that of other viral respiratory infections. For example, aerosols captured at the mouth of individuals with influenza, seasonal coronavirus, or rhinovirus infection contain viral genetic material in particles <5 μm and >5 μm in diameter (29, 59): Only influenza has been assessed for viability using the same experimental apparatus, which found that a minority of samples in the smaller size range were viable (71). Lindsley et al. (64) cultured influenza A virus from cough aerosol particles in the size range 0.3–8 μm in diameter among 41% (7 of 17) of infected participants, and viable virus was detected in one sample of fluid that had impacted in the aerosol sampling system and was expected to reflect virus emitted in cough aerosol particles larger than 10–15 μm in diameter. Influenza RNA is detected more frequently than viable virus in the aerosols emitted from influenza cases (63), which likely reflects the lower sensitivity of culture-based methods relative to culture-independent methods. Overall, these studies have found high levels of interindividual variability in respiratory virus emission and affirmed that infected cases do not always emit detectable virus in respiratory aerosol.

### Pathway

The primary pathway of aerosol transmission involves the movement of infectious aerosol in air from the source to the receptor. This pathway requires (*a*) the pathogen to remain infectious, and (*b*) the pathogen to remain suspended in air long enough to reach the receptor. The transport of aerosol through air is a complex process influenced by particle size, initial velocity, and airflow patterns, among other factors (36); respiratory droplets also undergo dynamics owing to water evaporation (61). Thus, the event that causes an infectious particle to move through the air to reach a susceptible person is highly dependent on the setting and environmental conditions and may not be observed in all settings.

Experimental studies have demonstrated that SARS-CoV-2 aerosolized in respirable particles remains infectious for hours (half-life, *t*_1/2_ ∼1 h), though inactivation is rapidly increased with exposure to sunlight (99, 111). Studies have not been conducted with larger particles to date. The timescale of hours is long enough for HCWs to be exposed during patient care, which is affirmed by the detection of viable SARS-CoV-2 in air inside and outside patient rooms (57, 97).

With respect to particle transport, the routine detection of SARS-CoV-2 RNA in air within and beyond patient rooms, including on the surfaces of air ventilation grates (12), is consistent with broad dispersion through air. Furthermore, SARS-CoV-2 RNA has been shown to reach the breathing zones of HCWs, where it can be inhaled (97).

Few studies have considered the transport of other respiratory viruses through air in health care settings. Phan et al. (85) measured influenza, rhinovirus, and several other respiratory viruses in the air around hospitalized, infected patients. The concentration of virus gene copies in air varied widely, but the concentrations of different virus types were similar among the particle size bins (<1 μm, 1–4 μm, and >4 μm). Viruses were detected less frequently in the breathing zones of HCWs than at fixed locations in patient rooms, though this was likely an artifact of method sensitivity as the personal samples had a much shorter sampling duration than stationary samples. Furthermore, Thompson et al. (105) found H1N1 (in 2009) influenza RNA in 14% of air samples collected around hospitalized patients, but samples were positive for only 26% of patients studied.

An alternative pathway involves the resuspension of virus from fomites, where resuspended virus may be inhaled by susceptible individuals. Dust, which may contain microorganisms, can be resuspended from surfaces by normal human activities (88). Immune guinea pigs with influenza virus applied to their coats were able to transmit infection to susceptible guinea pigs in the absence of direct contact, suggesting transmission via virus aerosolized from fomites (7). It is not clear, however, how relevant this pathway is for SARS-CoV-2 and other respiratory viruses among humans.

### Receptor

To initiate infection, a virus must reach sites within the susceptible host where it can bind and enter host cells. Host cell binding and entry are mediated by the spike glycoprotein on the SARS-CoV-2 envelope, which binds to the angiotensin-converting enzyme 2 (ACE2) of the host cell. Virus binding with ACE2 and other host cell proteins, including the transmembrane protease serine-2 (TMPRSS2), begins membrane fusion and entry of the viral genome into the host cell (21). ACE2 and TMPRSS2 are distributed throughout the respiratory tract, from the nares to the alveolar region of the lung, enabling active viral replication throughout the respiratory tract (39, 41, 56, 114). Thus, SARS-CoV-2 may initiate infection in many locations in the respiratory tract, with the site dependent on the aerodynamic diameter of the inhaled virus-laden particle (36). This process has been observed among Syrian hamsters exposed to SARS-CoV-2, where replication was detected in the trachea and lung of hamsters inoculated by the inhalation of aerosol and in the epithelium of the nasal turbinates after exposure by intranasal instillation (87).

With respect to droplet spray, this route is plausible because SARS-CoV-2 infection can be initiated in the nasal epithelium (39). Though the mucociliary escalator clears material from the respiratory tract, moving virus upward away from the lungs into the oral cavity (82, 90), oral–lung aspiration can move virus from the oropharynx to the lungs (39). Thus virus deposited on the facial mucous membrane via droplet spray may reach the respiratory tract.

Limited research has described the magnitude and frequency of SARS-CoV-2 exposure at the receptor. Santarpia et al. (97) quantified SARS-CoV-2 RNA in personal air samplers worn by HCWs during patient care. The air sampling device used in this study, the Button Sampler, measures inhalable particles and thus may have captured virus projected via droplet spray or suspended in air (20). Ong et al. (81) sampled the personal protective equipment (PPE; goggles, front of respirator, and front of shoes) of 30 HCWs exiting the rooms of COVID-19 patients, but the virus was not detected in any of the 90 samples. A prior study by Ong et al. (79) reported that one swab collected from the front of a shoe worn by a HCW leaving a COVID-19 patient room was positive.

With respect to other respiratory viruses, Phan et al. (84, 85) found endemic respiratory viruses in the breathing zones of HCWs who were providing care to infected patients as well as on HCWs’ bodies, clothing, and PPE. It is not well understood how HCWs’ bodies, clothing, and PPE become contaminated with viruses, but contamination could be from the projection or deposition of aerosols or from contact with fomites.

### Epidemiologic Evidence

Two outbreaks early in the COVID-19 pandemic that were influential in supporting the role of aerosol transmission were the Skagit Valley Chorale outbreak and a restaurant outbreak in China. The Chorale outbreak occurred among 61 persons who attended a 2.5-h choir practice. The US CDC concluded, consistent with the transmission routes recognized by the organization at the time, that there were many opportunities for droplet transmission or fomite transmission, enhanced by the act of singing (33). A subsequent modeling analysis indicated that insufficient attendees were in close contact to experience droplet spray from the index case or to touch a common fomite, which allowed investigators to infer that a mean emission rate of 970 quanta per h could explain the observed infection rate (70). The restaurant outbreak in China involved secondary cases among customers in different parties seated at different tables from the index case, where the pattern of secondary cases was consistent with air conditioning airflow and could not be explained by fomites shared with the index case (66). Additional COVID-19 outbreaks are reviewed for evidence of aerosol transmission elsewhere (104).

### Knowledge Gaps

One key gap that persists with respect to the evidence base of aerosol transmission of COVID-19 and other viral respiratory infections is the distribution of viable virus across respiratory aerosol particles of different sizes. This information is very important because it determines how the virus will be transported through the environment and where it can deposit in the receptor. A barrier to filling this gap is aerosol sampling technology. Although the number and size of respiratory aerosols with d_a_ ≤30 μm can be measured using a number of methods (23), there is a gap in our ability to capture particles of specific sizes (e.g., 10–15 μm) and detect viruses in nonrespirable particles. Table 2 summarizes common aerosol sampling methods used to detect respiratory viruses. Notably, none of the methods provide size-specific ascertainment of viruses for particles greater than 5–10 μm in diameter. A more general related issue, which affects interpretation of SARS-CoV-2 air sampling data for risk assessment, is the lack of information about sampler performance with respect to detection limits and sampler capture efficiency by particle size (24).

A second key gap is the process by which virus that deposits on the facial mucous membranes moves inside the body to sites where infection can be initiated, as well as the efficiency of this process. The fate of virus-laden particles that deposit on the lips, nares, or eyes is important because it influences how much droplet spray (and indirect contact transmission) can contribute to infection risk. SARS-CoV-2 that deposits in the eye can reach the nasal and nasopharyngeal mucosa (103), which is also observed for influenza (10, 14). Hou et al. (39) has suggested oral–lung aspiration as a mechanism that can move virus from the oropharynx to the lungs. Virus that deposits on the lip or in the mouth may be ingested, and though SARS-CoV-2 can replicate in the gastrointestinal tract, infection at that site is thought to be secondary to primary infection in the respiratory tract (44).

## EVIDENCE FOR INDIRECT CONTACT TRANSMISSION

### Source

Viruses in respiratory secretions of an infected person are thought to be introduced to fomites by the deposition of infectious aerosol and direct contamination by infectious respiratory secretions, such as by wiping one’s mouth with a hand or tissue. As previously described, viable SARS-CoV-2 is present in respiratory secretions (114), and infectious aerosol is emitted from COVID-19 cases. SARS-CoV-2 RNA is commonly found on surfaces around hospitalized COVID-19 patients; viable virus has been rarely detected (1, 11, 26, 72). This evidence indicates that COVID-19 patients shed viable SARS-CoV-2 into their environment, contaminating fomites.

### Pathway

A commonly discussed pathway for the indirect contact transmission of respiratory viruses involves deposition of infectious aerosol onto a surface, virus transfer to the hand of the HCW upon contact with the fomite, and virus transfer to the facial mucous membranes of the HCW when touched by their own hand (101) (Figure 1). This pathway requires (*a*) the pathogen to remain infectious on the fomite(s) and (*b*) the pathogen to be transferred with a nonzero efficiency between fomites upon contact.

Experimental studies have demonstrated that SARS-CoV-2 can remain viable on a fomite on the order of hours (*t*_1/2_ = 1–6 h), depending on the surface material (51, 111). SARS-CoV-2 inactivation is accelerated on fomites by increased temperature and relative humidity (13). On cadaver skin, SARS-CoV-2 has been found to have a *t*_1/2_ of 2–4 h (38). This timescale of inactivation is such that SARS-CoV-2 can remain infectious long enough on fomites to reach a receptor.

SARS-CoV-2 RNA is ubiquitous on fomites around COVID-19 cases in health care facilities, but viable SARS-CoV-2 has been infrequently detected (1, 11, 26, 72, 116). The infrequency of viable SARS-CoV-2 detection on fomites may be a result of the sensitivity of sampling and culture-dependent analyses rather than an absence of infectious virus (50, 116). The presence of SARS-CoV-2 RNA on surfaces is generally correlated with presence in air (24).

The efficiency of SARS-CoV-2 transfer between fomites such as during hand-to-surface or hand-to-facial mucous membrane contacts has not been thoroughly studied to date. Todt et al. (107) demonstrated that touching a coin or banknote with artificial skin removed SARS-CoV-2 from the item, indicating virus loss with contact. Daller et al. (27) observed that human coronaviruses 229E and OC43 did not transfer from fingertips to surfaces upon contact, but OC43 was transferred to steel, apple, and cucumber when fecal material was included in the matrix, with varied efficiency among the surfaces. Prior work in this area has demonstrated that there is substantial variability in transfer efficiency among viruses and that this process is influenced by the matrix, substrate, direction of transfer, and environmental conditions (4, 5, 65, 109). Overall, the transfer of SARS-CoV-2 between fomites likely occurs, though the efficiency of this transfer is not well understood at this time.

### Receptor

HCWs touch their facial mucous membranes (28, 54, 83). As described in the context of aerosol transmission, host cell receptors for SARS-CoV-2 are present in nares or accessible via the eyes and thus are accessible to virus deposited on the facial mucous membranes via indirect contact transmission.

SARS-CoV-2 infection has been demonstrated in Syrian hamsters exposed to virus on fomites (cages previously occupied by infected hamsters), with antigen detected in the ciliated epithelium of the nasal turbinates but not in the olfactory epithelium, trachea, or lungs; these fomite-exposed animals exhibited delayed-onset and less severe disease than did animals exposed to SARS-CoV-2 aerosol (87). While the patterns of virus replication and antigen expression in these animals are suggestive of contact rather than inhalation exposure, aerosolization of SARS-CoV-2 from fomites may have occurred (7).

### Epidemiologic Evidence

There is little epidemiological evidence to support the indirect contact route for SARS-CoV-2 transmission, in part because of the simultaneous potential for aerosol transmission. In an investigation of an apartment building outbreak in Guangzhou, China, however, Xie et al. (115) attributed transmission between two families to contact with an elevator button, though SARS-CoV-2 RNA was not found on the button in subsequent environmental testing.

### Knowledge Gaps

One key gap that persists with respect to the evidence base for indirect contact transmission is how much virus actually reaches the facial mucous membranes through contact and the efficiency with which the virus moves to sites in the body where infection can occur. To our knowledge, pathogens have not been measured on the facial mucous membranes of HCWs or other people. Phan et al. (84) found seasonal influenza virus on the cheeks of 2 of 21 HCWs after providing care to an influenza patient, suggesting that virus may also be present on the facial mucous membranes.

A second gap relates to characterizing the process of virus shedding onto fomites. While we know that respiratory viruses are present in respiratory fluids and on fomites around infected patients, there is a lack of data with which to develop the pathway. For example, the contribution of virus ejected by coughs and sneezes relative to gains and losses due to hand-to-fomite contacts is unknown. These relative contributions are likely scenario specific and influenced by the proximity of cases to fomites, the symptoms and shedding by a case, and human contact behaviors.

## ROLE OF QUANTITATIVE MODELING

Quantitative modeling is used herein to describe mathematical representations of the mechanisms by which virus moves from the source, through the environment, to the receptor; the dose, the amount of virus that reaches a receptor, is used to calculate the probability of infection. Quantitative modeling is therefore the exposure assessment step in the framework of quantitative microbial risk assessment (31). One strength of quantitative modeling methods for the study of infectious disease transmission is that they require explication and quantification of the mechanisms hypothesized to be involved, which opens the transmission process for interrogation through logic, experiment, and observation. Another strength is the ability of models to explore scenarios that may not be observed. Some examples of quantitative models of COVID-19 transmission are provided to highlight how these methods can identify key determinants of infection risk, as well as knowledge gaps.

Jones (45) modeled the relative contributions of inhalation, droplet spray, and indirect contact transmission to the risk of COVID-19 infection among HCWs during patient care and found that the three routes contributed 30%, 60%, and 1.4% of the infection risk, on average, in the absence of PPE. PPE use was found to influence both the overall risk of infection and the relative contributions of each transmission route to the risk of infection. The contribution of inhalation exposure to risk was found to be strongly positively influenced by the amount of virus emitted in respirable particles. There remains, however, a knowledge gap about the distribution of viable SARS-CoV-2 and other viruses among respiratory aerosols of different sizes.

Azimi et al. (8) modeled the COVID-19 outbreak on the *Diamond Princess* cruise ship and found that close-range (droplet spray and inhalation), long-range (inhalation), and fomite-mediated transmission contributed similar numbers of cases but that inhalation of aerosols contributed more than droplet spray (59% versus 41%, on average). An interesting parameter that was explored in this model was the ratio of the median infectious dose (ID_50_) for SARS-CoV-2 in the upper and lower respiratory tracts (URT and LRT). The ID_50_ for SARS-CoV-2 is currently unknown for humans, but the possibility that ID_50_ varies between the URT and the LRT has been explored for influenza (74). Azimi et al. (8) initially considered URT:LRT ID_50_ ratios of 1:1, 10:1, and 100:1 and found that the outbreak could be reproduced using the model with all three of these ratios. It is not clear how to really interpret this finding, given the high variability in ratios that reproduced the outbreak. This gap extends to other respiratory viruses.

Miller et al. (70) modeled the Skagit Valley Chorale outbreak to demonstrate that the inhalation of aerosols could explain the outbreak. The model used was an adaptation of the Wells–Riley model of infection risk through airborne transmission. A mean emission rate of 970 quanta per h reproduced the observed outbreak. Quanta is an empirical, not biological, unit, but this modeling analysis highlights that the emission rate of SARS-CoV-2 is a key knowledge gap. Not only can the emission rate help explain outbreaks, but it can also be used with quantitative models to determine recommended ventilation rates and occupancy durations for occupied environments where COVID-19 cases may be present (18).

Several studies have modeled COVID-19 transmission through indirect contact involving single touches (34, 86, 113). These studies all found that infection risk is low in the scenarios studied, indicating that limited surface cleaning is needed to achieve de minimus risks, but these scenarios are highly simplified. Human contact behaviors are highly complex and variable (83), and there is a gap in knowledge about the frequency and sequence of contacts with fomites among HCWs and others. Furthermore, the sparse data about viable virus on surfaces (and in air) require assumptions about the ratio of gene copies to viable virus. A 1:1 ratio is generally considered unlikely—gene copies are typically more numerous than viable virus, but the ratio has been found to vary based on analysis methods (17, 96). This relationship is a key knowledge gap for quantitative modeling and risk assessment.

## INTERVENTIONS

Effective interventions interrupt the transmission process by (*a*) preventing or containing emission at the source, (*b*) interrupting the environmental pathway, or (*c*) preventing exposure and infection among susceptible receptors. Each will be discussed in turn, with specific focus on the transmission of COVID-19 from patients to HCWs.

### Source

Emission of infectious aerosols from the source may be limited by face coverings. Lindsley et al. (62) found that a medical-grade procedure mask, a three-ply cotton cloth face mask, and a single- or double-layer polyester neck gaiter blocked 50–60% of aerosol particles (d_a_ ≤7 μm) emitted from a head form by a cough simulator. Leung et al. (59) found that surgical face masks reduced the emission of seasonal coronavirus and influenza, but not rhinovirus, from cases, but the effect differed by particle size among the viruses. Epidemiologic studies of face coverings as source controls have been hindered by noncompliance, but some studies have reported reduced infection rates among household contacts of cases who wear face coverings (68).

The COVID-19 pandemic led to a plethora of barrier devices intended to prevent infectious respiratory aerosols from contaminating the health care environment and HCWs (102). Some of these devices are simply physical barriers to prevent the splash or spray of aerosol onto surfaces and HCWs, such as the iconic acrylic box design by Lai (55). Other devices include local exhaust ventilation (35). Few studies, however, have rigorously evaluated the user experience or aerosol capture efficiency of these devices, and initial efforts indicate a need for continued user-centered design to improve usability and performance (102). Previously, ventilated bed headboards and filtration units had been developed to capture respiratory aerosols from patients in temporary hospitals (69): Though effective, these designs have not been widely adopted during the COVID-19 pandemic.

### Pathway

With respect to the pathway, intervention strategies fall into three main categories: (*a*) ventilation and filtration of air, (*b*) physical distancing between sources and receptors, and (*c*) cleaning of fomites.

In health care settings, dilution ventilation is delivered through complex building heating, ventilating, and air conditioning (HVAC) systems (3, 98), which can be difficult to modify without substantial investment. Portable air filters have not been widely discussed to enhance air cleaning in health care facilities but have been found to reduce SARS-CoV-2 in air and on surfaces in households (94), if not the transmission of respiratory infections to date (32).

Physical distancing has been a key strategy in the prevention of COVID-19 transmission in the community and workplaces, leading to the implementation of occupancy limits, working from home, and signage indicating minimum distances. Physical distancing may be difficult in health care settings owing to job demands, such as multidisciplinary clinical rounds, and the design of clinical workrooms and call rooms (6). Creative ideas about the use of technology and the physical plant can facilitate physical distancing among HCWs, however, as in other workplaces.

Cleaning and disinfection of fomites and hand hygiene have been core public health messages during COVID-19 but were already common in health care (101). The US Environmental Protection Agency has approved disinfectants for use with SARS-CoV-2, though few have been specifically tested with SARS-CoV-2 (110). Alcohol-based disinfectants (≥40% ethanol or ≥70% isopropyl alcohol) are effective at inactivating SARS-CoV-2, including on human skin (37, 53), motivating widespread use.

### Receptor

Vaccines are the surest way of protecting receptors from infection. COVID-19 vaccines currently authorized for use in the United States have demonstrated high efficacy at preventing symptomatic, laboratory-confirmed COVID-19 infections (76, 77, 78). A network of longitudinal cohort studies of HCWs, first responders, and other essential and frontline workers across eight locations in the United States found vaccine effectiveness of full immunization with two doses of mRNA vaccines to be 90% [95% confidence interval (CI) 68–97%] against laboratory-confirmed SARS-CoV-2 infection (106).

In health care settings, PPE is a common infection prevention strategy, where the choice of PPE depends on the infectious disease (101). The debate has been not whether HCWs should wear PPE while caring for COVID-19 patients, but whether they should wear respirators or surgical masks. The answer depends on whether one believes that COVID-19 can result from the inhalation of SARS-CoV-2 or only from deposition onto the facial mucous membranes. Both surgical masks and respirators serve as barriers to the facial mucous membrane deposition, whereas only respirators effectively prevent the inhalation of airborne contaminants, including viruses. Table 3 summarizes the major categories of respirators. Globally, a wide variety of respirators and face coverings have been used in health care, and their testing and performance standards are reviewed elsewhere (47).

The receptor has an important role in indirect contact transmission, as their hand-to-face contacts may transfer virus onto the facial mucous membranes. Among research workers in biosafety level 2 laboratories, hand-to-face contact frequency was modestly influenced by perceived risk (43), suggesting that education could effect behavior change. The effectiveness of face coverings and PPE at reducing hand-to-face contacts or transfer of virus to facial mucous membranes has not been assessed to date.

### Knowledge Gaps

The hierarchy of controls in occupational health prioritizes the use of engineering controls to contain infectious aerosols at the source, the infectious patient. As a result, it is appropriate to prioritize the development and evaluation of aerosol containment devices that can provide respiratory isolation for patients during routine care and patient transport and to capture aerosols generated during medical procedures. Early work with these devices suggests feasibility, but there remains a significant need for user-centered design and evaluation to ensure that these devices meet user needs and effectively control infectious aerosols of diverse sizes (102). Portable air filtration is less valuable in health care settings because it affects the pathway rather than the source and can take up space in patient rooms. In health care, such devices may be more effective for temporary emergency facilities (69).

Though PPE is considered the last resort in the hierarchy of controls, the value of respirators, surgical masks, and other face coverings for source control is a critical knowledge gap. Owing to the prevalence of asymptomatic COVID-19 infections, concerns were raised that HCWs may shed SARS-CoV-2 through respirator exhalation valves and air flowing out of powered air-purifying respirator (PAPR) hoods or helmets. Aerosols follow a circuitous route to pass through an exhalation valve or from a hood or helmet, which can cause particle deposition and reduce emission (16). Nonetheless, there is a new recognition of the need to understand how respirators, particularly PAPRs and respirators with exhalation valves, function as a source control. In contrast with respirators, surgical masks were originally designed for source control to protect the surgical field from respiratory aerosols emitted by HCWs. Despite this origin, the data about the source control offered by surgical masks remain deficient. As found by Leung et al. (59), surgical masks may have inconsistent performance as source control.

Two other knowledge gaps about controls for SARS-CoV-2 that warrant further research, but are less likely to transform control strategies for viral respiratory infections, include (*a*) the trade-off in risk between the reduction in microbial contamination from increased environmental cleaning in disinfection and adverse health outcomes among HCWs from increased exposures to these products, and (*b*) the effect of PPE on hand-to-face contact and exposure through the indirect contact transmission route. With respect to the first topic, Quinn et al. (89) have proposed an integrated framework for infection and occupational illness prevention associated with cleaning and disinfection agents. With respect to the second topic, hand-to-face contacts may be influenced by perceptions of risk (43), while PPE may alter hand-to-face contact behavior and serve as barriers to deposition upon contact. There remains, however, a lack of knowledge about how to effectively change hand-to-face contact behaviors and how effective various types of PPE, such as surgical masks or respirators, are as barriers to pathogen deposition.

## FUTURE DIRECTIONS

Greater consensus has emerged about the role of aerosols in the transmission of COVID-19 over the first year of the pandemic, notably marked by acknowledgment by both the WHO and the US CDC of the role of aerosol inhalation, in addition to droplet spray, in COVID-19 transmission (19, 112). The next step is for these and other organizations to update guidelines and practices accordingly: specifically, to recommend forms of PPE that effectively and meaningfully reduce the risk of inhaling SARS-CoV-2 aerosols—i.e., respirators. HCWs in California are protected by the requirement that respirators, not surgical masks, be used when HCWs may be exposed to aerosol-transmissible diseases (Calif. Code Regul. Title 8, §5199). The US Occupational Safety and Health Administration (OSHA) has issued an Emergency Temporary Standard for COVID-19 that applies to health care settings and requires that respirators and other PPE be provided to employees who have exposure to a person with suspected or confirmed COVID-19, including during aerosol-generating procedures (75).

COVID-19 is unique from other recent emerging and endemic viral respiratory infectious diseases only in its global toll on health and well-being. As research continues to illuminate the transmission processes of respiratory viruses, more similarities than differences are apparent among viruses, and more weaknesses of the current paradigm of contact, droplet, and airborne transmission emerge. Jones & Brosseau (46) found strong evidence that seasonal influenza, SARS, norovirus, and Ebola virus are aerosol-transmissible diseases. For some diseases, the role of aerosol transmission may be opportunistic (95), but that does not necessarily mean that the transmission pathway is negligible. It is time to update our disease transmission paradigm to reflect the current knowledge generated through multidisciplinary and interdisciplinary research, particularly with reference to the role of aerosols in the transmission of infectious diseases.

A challenge for preventing the transmission of viral respiratory diseases, including COVID-19, is the lack of consensus about what is an acceptable level of risk for HCWs, for workers in other settings, or for those in the general public. The acceptable level of risk is what drives the setting of limits for exposure limits, and it is exposure limits that permit one to evaluate whether the amount of virus in the environment is acceptable to permit occupancy or require additional controls. Such a limit is further complicated by our current lack of knowledge about the infectivity of SARS-CoV-2 and many other respiratory pathogens. Infectivity is best described by a dose–response function that relates the probability of a response (typically infection) in a susceptible host for a given dose of pathogen. While infectivity studies in animals can be helpful, there is uncertainty about extrapolation from animals to humans, and many studies do not involve exposure routes relevant for human-to-human disease transmission (30). Despite ethical concerns, human challenge studies with SARS-CoV-2 are in development, which can inform vaccine development and infectivity through different routes of exposure (52).

## CONCLUSIONS

Strong evidence indicates that COVID-19, like other viral respiratory infectious diseases, is an aerosol-transmissible disease. The most pressing research question with respect to understanding the aerosol transmission is the emission of viable viruses in respiratory aerosols of different particle sizes, as the size of virus-laden particles influences where the virus is likely to deposit and initiate infection in the respiratory tract. Such research should be conducted for multiple respiratory viruses because aerosol emission and infection risk likely vary between viruses. With respect to preventing aerosol transmission, research should be prioritized to improve source controls, including devices to contain respiratory aerosols generated by patients and medical procedures. In conjunction, infection prevention guidelines need to be updated so that they reduce aerosol exposures among HCWs effectively; such updates should include recommendations and regulations for the use of respiratory protection. No one likes to wear a respirator, but the goal is to improve the user experience through respirator design and not to opt for inadequate protections for HCWs.

Source control strategies and PPE will reduce the potential for indirect contact transmission of COVID-19 and other viral respiratory infectious diseases to HCWs by reducing environmental surface contamination and providing barrier protection for HCWs. Research about how and how efficiently virus that deposits on the facial mucous membranes through contact and droplet spray reaches sites where infection can be initiated, which likely varies among viruses, would help to illuminate how significant the indirect contact (and droplet spray) transmission route is for different respiratory viruses. Such research could take a mechanistic approach or involve infectivity studies with exposure through droplet spray or contact deposition onto the facial mucous membranes.

## Figures and Tables

**Table 1:** Source–pathway–receptor framework for assessing disease transmission processes for viral respiratory infections

Component	Fundamental question
Source	How are pathogens emitted from an infectious person?
Pathway	Can pathogens remain infectious and move through the environment in air or on fomites from the source to a receptor?
Receptor	How do pathogens reach sites within a susceptible person where infection can be initiated?

**Table 2:** Common aerosol sampling methods used to detect respiratory viruses, with selected references for studies that have used this method for severe acute respiratory syndrome coronavirus 2 (SARS-CoV-2) sampling

Method	Description
Anderson cascade impactors	Operates at high airflow rate and impacts aerosols onto a plate. Particles are size-separated from >9 μm to <0.3 μm, depending on the number of plate stages.
National Institute for Occupational Safety and Health two-stage BioSampler	Operates at 3.5 liters per min and separates aerosols into three size fractions. Particles with diameter >4.1 μm are captured on a filter, while particles with diameter 1–4.1 μm and <1 μm are sampled into tubes. Can be used for personal sampling (25).
Closed-face filter cassette	Operates at 2 liters per min and collects particles approximately <30 μm in diameter onto a filter. Can be used for personal sampling.
BioSpot-VIVAS	Operates at 11 liters per min and condenses water onto the aerosol to deposit into a petri dish or other receptacle (58).
Button sampler	Operates at 4 liters per min and collects the inhalable particles (<100 μm) onto a gelatin or other type of filter. Can be used for personal sampling (97).
MD8 portable air sampler	Operates at 50 liters per min. Aerosols are collected onto a gelatin filter (92, 97). Size range of measured aerosols unknown. Handheld device.

**Table 3:** Major categories of respirators used in health care, and references with examples of studies about their use to prevent COVID-19 among health care workers

Category	Description
Powered air-purifying respirators (PAPRs)	Head gear varies but typically covers the face and head as a hood/shroud or helmet with visor. A fan delivers filtered air into the head gear.
Elastomeric respirators	Facepiece is rubber and covers half the face (nose and mouth) or the full face with integrated visor. Air breathed in by the wearer passes through filter cartridges fitted to the facepiece. Facepiece has a valve that releases the exhaled breath of the wearer.
Filtering facepiece respirators or filtering face masks	Facepiece is filter material and covers half the face (nose and mouth). Some models have a valve that releases the exhaled breath of the wearer.

## SUMMARY POINTS

1. COVID-19 is an aerosol-transmissible disease similar to other viral respiratory infectious diseases.
2. Aerosol transmission likely contributes more to COVID-19 transmission than does indirect contact transmission, though the relative contributions may vary among other respiratory viruses and exposure scenarios.
3. Recommendations and regulations about disease transmission must be updated to better protect HCWs from aerosol-transmissible respiratory infectious diseases, such as COVID-19.
4. Mechanistic frameworks, including quantitative models, require infectious disease transmission processes to be made transparent, enabling them to be interrogated through logic, experiment, and observation.
5. While knowledge gaps remain about how COVID-19 is transmitted, there is sufficient information to take actions to prevention transmission, including through broader use of respirators and use of source controls to prevent infectious aerosol emission from patients.

## FUTURE ISSUES

1. What is the distribution of viable viruses in respiratory aerosol particles of different sizes?
2. How does virus on the facial mucous membranes move to reach sites inside the body where infection can be initiated?
3. How effective are face coverings to reducing emission of infectious respiratory aerosols?
4. Can usable, effective aerosol containment devices be developed to prevent emission of infectious respiratory aerosols from patients during normal activities and medical procedures?
5. How infectious are SARS-CoV-2 and other respiratory viruses through different exposure processes?
6. What is an acceptable risk of infection for HCWs?

## Abbreviations

Aerosol: a suspension of droplets or solid particles in a gas, without reference to size
Aerosol transmission: when an infectious aerosol enters the respiratory tract or is projected onto the facial mucous membranes of a susceptible person
Indirect contact transmission: when pathogens are transmitted from an infected person to a susceptible person via a contaminated intermediate object
Droplet: a liquid particle of any size
Short-range airborne transmission: when infectious aerosols in the respirable size range are inhaled by a susceptible person while in close proximity
Airborne transmission: when an infectious aerosol with respirable particles is inhaled by a susceptible person >6 feet from the source
Fomite: a surface or object capable of being contaminated with a pathogen
Particle: a small portion of matter
Respirable particles: particles of a size that can penetrate to the unciliated airways when inhaled, generally <10 μm in diameter
Infectious aerosol: an aerosol of infectious agents
Droplet spray (exposure): when droplets from the respiratory tract project onto the facial mucous membranes of a susceptible person
Droplet transmission: droplet spray exposure with infectious aerosols >5 μm in diameter and a nearby (<3 feet) susceptible person
Inhalable particles: particles of a size that can enter into the nose and/or mouth during breathing, generally <100 μm in diameter
